# Advances in imaging findings of preeclampsia-related reversible posterior leukoencephalopathy syndrome

**DOI:** 10.3389/fnins.2023.1144867

**Published:** 2023-03-31

**Authors:** Nan Zhang, Linfeng Yang, Aiqing Han, Yuanyuan Wang, Guiwu Zhao, Yue Wang, Tao Chen

**Affiliations:** ^1^Department of Radiology, Shandong Provincial Hospital Affiliated to Shandong First Medical University, Jinan, Shandong, China; ^2^Department of Radiology, Jinan Maternity and Child Care Hospital Affiliated to Shandong First Medical University, Jinan, Shandong, China; ^3^Department of Obstetrics, Jinan Maternity and Child Care Hospital Affiliated to Shandong First Medical University, Jinan, Shandong, China; ^4^Department of Radiology, Binzhou Medical University, Yantai, Shandong, China; ^5^Zhucheng Peace Medical Imaging Diagnosis Center, Weifang, Shandong, China; ^6^Department of Clinical Laboratory, Jinan Maternity and Child Care Hospital Affiliated to Shandong First Medical University, Jinan, Shandong, China

**Keywords:** preeclampsia, reversible posterior leukoencephalopathy syndrome (RPLS), neuroimaging, magnetic resonance imaging, multimodal brain MRI

## Abstract

Preeclampsia (PE)-related reversible posterior leukoencephalopathy syndrome (RPLS) is a common complication of hypertensive disorders of pregnancy. The syndrome usually occurs after 20 weeks of gestation and can lead to brain injury. Severe headache, seizures, disturbance of consciousness, and other neurological symptoms may occur in severe cases. PE-RPLS has high morbidity and mortality rates and seriously damages maternal and fetal health. In recent years, the continuous advancement of medical imaging technology has provided an important imaging basis for the early diagnosis and prognostic evaluation of RPLS. This article mainly details the research status of the etiology and pathogenesis of PE-RPLS and describes its characteristic imaging findings, especially MRI findings, to provide new insights into its early diagnosis, early treatment, and improvement of prognosis.

## 1. Introduction

Hypertensive disorders of pregnancy (HDP) are some of the most common gestational cardiovascular diseases. Worldwide, the incidence rate of HDP is as high as 10%. A number of domestic and foreign guidelines classify HDP into four types, namely, preeclampsia (PE), chronic hypertension, chronic hypertension with PE, and gestational hypertension, according to the onset time of hypertension, the presence or absence of proteinuria, other target organ damage, and clinical manifestations (Wenger et al., 2018). PE is the most common and serious type of HDP. It manifests as elevated blood pressure after 20 weeks of gestation accompanied by different degrees of end-organ damage [such as increased liver alanine transaminase (ALT), decreased platelet count (PLT), and renal insufficiency], with or without proteinuria. PE with seizure is called eclampsia.

With the gradual liberalization of the three-child policy in China, the number of pregnancies at advanced maternal age has increased significantly, and the incidence rate of PE has increased significantly. PE is one of the pregnancy complications that obstetricians are most worried about (Ananth et al., 2013). This disease has high morbidity and mortality rates and seriously threatens the maternal and fetal life and health. PE usually affects multiple systems throughout the body, especially the central nervous system, and reversible posterior leukoencephalopathy syndrome (RPLS) is the most common complication of the central nervous system of PE.

This article reviews the advances in central nervous system imaging of PE-related RPLS based on the etiology and pathogenesis of the disease, especially the advances in MRI for PE-related RPLS, aiming to provide a theoretical basis for the early detection, early diagnosis, and early treatment of the disease.

## 2. The etiology and pathogenesis of PE-related RPLS

Reversible posterior leukoencephalopathy syndrome can be caused by a variety of etiologies, including PE, renal insufficiency, elevated blood pressure, component transfusions, autoimmune disease, infection, and shock (Tlemsani et al., 2011; Fa’ak et al., 2021; Virojtriratana et al., 2022). PE is one of the common causes of RPLS, and RPLS is more likely to occur in PE patients with severe headaches, consciousness impairment, visual abnormalities, or other neurological symptoms (Demirtaş et al., 2005; Murali et al., 2020). Eclampsia is an acute cerebrovascular complication considered a clinical manifestation of RPLS (Damak et al., 2022; Fishel Bartal and Sibai, 2022).

No consensus has been reached on the pathophysiological mechanism of PE-related RPLS, but many studies have noted that it may be related to the increase in vascular permeability caused by vascular endothelial injury and the dysfunction of vascular autoregulation caused by excessively high blood pressure. Under normal circumstances, although the cerebral perfusion pressure fluctuates within the range of approximately 50–150 mmHg, the cerebral circulation maintains a constant cerebral blood flow, and the process of maintaining a constant cerebral blood flow through the dilation and contraction of small blood vessels is called cerebral blood flow autoregulation. The blood supply to the back of the brain mainly comes from the vertebral-basilar system. However, since there are almost no sympathetic nerves in the vertebral-basilar system, the system (especially the cerebral blood vessels supplying the occipital lobe and the posterior watershed area) is quite sensitive to blood pressure fluctuations. When blood pressure rises rapidly and exceeds the upper limit of cerebrovascular autoregulation, the area supplied by the posterior cerebral artery is excessively perfused, the blood−brain barrier is destroyed, and a large amount of plasma and macromolecular substances extravasate, resulting in vasogenic cerebral edema (Fugate and Rabinstein, 2015; Virojtriratana et al., 2022). Therefore, the lesions of RPLS are mostly located in the area supplied by the posterior cerebral artery, with a symmetrical distribution, mainly involving the cortex and subcortical white matter areas.

In addition, studies have shown that endothelial cell dysfunction caused by systemic toxic effects is a key step in the occurrence of PE-related RPLS. Cerebral hypoperfusion due to vasoconstriction may be responsible for the development of RPLS, and hypertension may be a response to cerebral hypoperfusion due to endothelial dysfunction caused by systemic toxic effects (Hawkes et al., 2022). Lactate dehydrogenase (LDH) is a marker of endothelial cell damage. Some studies found that PE-related RPLS is closely related to the LDH level. Capillary permeability increases due to vascular endothelial injury, and hypoalbuminemia reduces colloid osmotic pressure and induces the development of RPLS. As the condition improves, the LDH level quickly returns to normal. Some scholars believe that vasogenic cerebral edema in RPLS is caused by the inflow of intravascular fluid into the interstitium due to venous constriction–induced increases in capillary hydrostatic pressure and microvascular permeability (Singhal, 2021). However, this hypothesis does not explain the finding that hypertension often precedes the onset of RPLS symptoms (Rabinstein et al., 2012). Some studies have suggested that the ischemic lesions often caused by vasospasm are mostly located in the cortex or white matter near the cortex (Li et al., 2023). If not treated in time, the lesions will often progress to cytotoxic edema, that is, cerebral infarction. Significant blood pressure fluctuations may be more likely to cause RPLS than increases in absolute blood pressure (Liman et al., 2014), and even septic and hypotensive patients may develop RPLS (Bartynski et al., 2006). The mean baseline blood pressure and the proportion and speed of blood pressure rise may be important factors in the disruption of the blood−brain barrier and the generation of vasogenic edema in RPLS patients.

## 3. Studies on central nervous system imaging of PE-related RPLS

The concept of RPLS was first proposed by Hinchey et al. (1996). In the past two decades, with the development and application of various imaging techniques, an increasing number of PE-related RPLS cases have been discovered and reported. The most common imaging methods for PE-related RPLS are CT and MRI (Table 1).

**TABLE 1:** Features on CT and MRI imaging with posterior leukoencephalopathy syndrome (RPLS).

Types	Features on CT and MRI imaging
Regions	Mainly occur in bilateral parieto-occipital lobes;
	Often involve the frontal and temporal lobes;
	The corpus callosum, thalamus, basal ganglia, cerebellum, or brainstem are rarely affected.
Density/Signal intensity	Density: low density.
	T1-weighted images: iso-intense or low signal intensity;
	T2-weighted images and FLAIR: high signal intensity;
	Diffusion-weighted imaging (DWI): isointense or hypointense;
	Apparent diffusion coefficient (ADC): hyperintense.
Contrast enhancement	Not usually seen after injection of a contrast agent.
Characteristics of edema	Almost involving both sides;
	Not completely symmetrical appearance.
Concomitant signs	Intraparenchymal hemorrhage; subarachnoid hemorrhage (SAH).

### 3.1. Application of CT to PE-related RPLS patients

CT has high-density resolution and unique advantages in diagnosing intracranial hemorrhage. However, there are no specific CT signs for different types of cerebral edema. Most RPLS lesions on CT show symmetrically distributed, patchy hypodensities in the bilateral parietal and occipital parenchyma, which may have a mass effect, but there may be no discernible abnormality on early CT examination. CT involves ionizing radiation, which brings a certain risk of teratogenicity, and has poor soft tissue resolution and poor visualization of small lesions, which limits its wide application in pregnant patients.

### 3.2. Application of MRI in PE-related RPLS patients

Magnetic resonance imaging has clear soft tissue contrast, high spatial resolution, and no ionizing radiation and so has become the preferred imaging method for diagnosing RPLS (Kastrup et al., 2015). Conventional MRI examinations include T1-weighted imaging (T1WI), T2-weighted imaging (T2WI), and T2-fluid-attenuated inversion recovery (FLAIR) sequences. When brain lesions are found in conventional MRI examinations, diffusion-weighted imaging (DWI) should be performed to identify the lesion types based on DWI and apparent diffusion coefficient (ADC) maps. When necessary, magnetic resonance angiography, magnetic resonance venography, and susceptibility-weighted imaging (SWI) techniques can be used to exclude cranial and cerebrovascular diseases. In addition, some emerging magnetic resonance techniques, including intravoxel incoherent motion (IVIM), quantitative susceptibility mapping (QSM), and magnetic resonance spectroscopy (MRS), have been applied to the study of brain function and metabolism in RPLS patients.

#### 3.2.1. Findings of conventional MRI sequences in patients with RPLS

**FIGURE 1:**
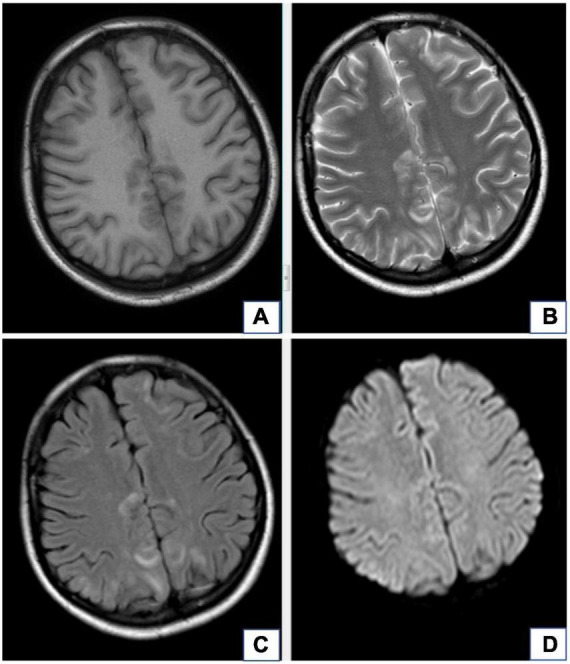
A 20-year-old female with diagnosis of preeclampsia-related reversible posterior leukoencephalopathy syndrome. The lesions occur in bilateral parieto-occipital lobes, which are isointense or hypointense on T1WI images **(A)**, hyperintense on T2WI images **(B)**, and hyperintense on T2-FLAIR **(C)**, but there is no obvious hyperintense signal in these lesions on DWI **(D)**.

Brain lesions in patients with RPLS mainly manifest as vasogenic edemas in the posterior white matter region on MRI, which are isointense or hypointense on T1WI images, hyperintense on T2WI images, and hyperintense on T2-FLAIR, and the lesions are often not enhanced much on scans by contrast medium. RPLS lesions mainly occur in bilateral parieto-occipital lobes, often involve the frontal and temporal lobes, but rarely involve the corpus callosum, thalamus, basal ganglia, cerebellum, or brainstem (Fischer and Schmutzhard, 2017; Figure 1). Umar et al. (2022)Please update the status of the references “Dong et al.” and “Hefzy et al.” and also include the same in the reference list. found that when the clinical symptoms of RPLS patients were headache, nausea, and vomiting, their brain imaging examinations had no specific findings, but when RPLS patients had seizures, their RPLS lesions were mainly distributed in the frontal cortex and parietal cortex. Fugate and Rabinstein (2015) believed that a distribution of RPLS lesions mainly in the posterior white matter region may be related to the nearly absence of sympathetic nerve distribution in this region and the susceptibility of this region to rapid blood pressure increases and cerebral hyperperfusion. Compared with the cerebral cortex, the white matter has a looser tissue structure, so the RPLS lesions are mostly located in the subcortical white matter region (the cortex is less likely to be involved).

Reversible posterior leukoencephalopathy syndrome lesions are not completely irreversible. Usually, they will improve spontaneously after early diagnosis and active treatment, and all patients have had improved clinical symptoms and MRI findings. Linear or gyrus-like enhancement was seen in approximately 20% of RPLS patients on contrast-enhanced MR images (Fugate et al., 2010; Gao et al., 2015; Kastrup et al., 2015), which may suggest the important role of endothelial dysfunction in the pathophysiological mechanism of RPLS. The pathophysiological mechanism of RPLS may be related to the endothelial dysfunction and the destruction of the blood−brain barrier caused by cytotoxicity or the inflammatory response.

Delayed treatment or the use of improper treatment methods may affect the condition of RPLS likely due to the increase in mean arterial pressure and the long persistence of pathogenic factors. Factors associated with poor outcomes include severe encephalopathy, a hypertensive cause, hyperglycemia, a neoplastic cause, a longer time to control the causative factor, the presence of multiple comorbidities, elevated C reactive protein, low cerebrospinal fluid glucose, and coagulopathy (Triplett et al., 2022).

Although its nomenclature is well known, many clinical researchers believe that the nomenclature of RPLS is inaccurate because cerebral edema is often not limited to the posterior white matter and is not always reversible. In mild cases, RPLS may only cause some clinical symptoms, such as headache or seizure activity. For these cases, the results of conventional MRI examinations may only show vasogenic edema in a small area or even no obvious abnormality. In some severe cases, brain MRI findings include acute intracranial hemorrhage and/or extensive edema of the posterior cranial fossa, which result(s) in obstructive hydrocephalus or compression of the brainstem, seriously endangering the mother’s life. After timely treatment in the early stage, the clinical symptoms can be significantly improved in most RPLS patients. Without delayed diagnosis and treatment, that may lead to mortality or irreversible neurological deficits (Parasher and Jhamb, 2020).

Sometimes it is necessary to distinguish RPLS from viral encephalitis. The lesions of viral encephalitis occur most commonly in the temporal lobe but can occur in any part of the brain, and most of them are unilateral hypodensities with a mass effect around them. The clinical symptoms of viral encephalitis include fever, headache, nausea, vomiting, and loss of consciousness. Electroencephalography and cerebrospinal fluid laboratory tests can provide a clear diagnosis.

#### 3.2.2. Diagnostic value of DWI for RPLS

Brain lesions in RPLS patients often appear isointense or hypointense on DWI but hyperintense on ADC maps. Some 15–33.3% of RPLS patients have focal restricted diffusion in the brain on DWI images, which is mostly distributed in punctate or patchy areas of angioedema and is related to the severity of edema. Large-area uniform restricted diffusion is rarely seen and is difficult to distinguish from ischemic infarction. Restricted diffusion may be associated with local edema, decreased perfusion, and vasospasm, suggesting irreversible structural damage and incomplete clinical recovery, although complete recovery of restricted diffusion or only mild atrophy after restricted diffusion has been reported in sporadic cases (Covarrubias et al., 2002; Yilmaz et al., 2010; Kastrup et al., 2015). Acute cytotoxic edema does not necessarily evolve into infarction. Up to now, restricted diffusion cannot be used to effectively assess the clinical prognosis of RPLS patients, and its potential value in assessing the clinical prognosis of RPLS patients still needs further research.

**FIGURE 2:**
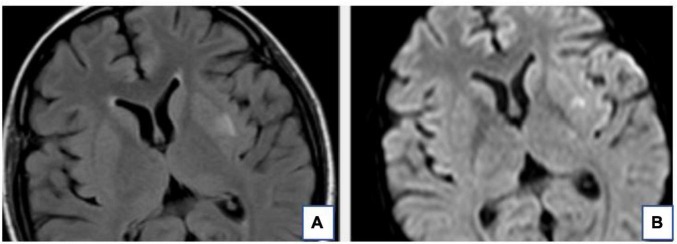
The T2-FLAIR **(A)** and DWI **(B)** of the same patient. The hyperintense signal of the Left basal ganglia area on T2-FLAIR is also very obvious on DWI. This suggests local infarction in the left basal ganglia.

The RPLS lesions of some patients are hyperintense on DWI and ADC maps, which means that the foci of hyperintensity on DWI probably reflects that they were affected by the T2 penetration effect. The RPLS lesions of some patients are hyperintense on DWI but hypointense on ADC maps, which indicates cytotoxic edema and poor prognosis (Figure 2.). DWI plays a key role in differentiating cytotoxic cerebral edema from vasogenic cerebral edema (Shankar and Banfield, 2017).

A recent study has shown that vascular endothelial injury plays an important role in the pathogenesis of RPLS and that cerebral edema on brain MR images of RPLS patients has a significant correlation with vascular endothelial injury, but no significant relationship with blood pressure fluctuations (Sundin and Johnson, 2018). MR images showed that the brain lesions were gradually disappearing after antispasmodic and antihypertensive treatments, which could also provide a basis for the theory of cerebral edema formation.

#### 3.2.3. The diagnostic value of SWI for RPLS

Susceptibility-weighted imaging is based on the differences in magnetic susceptibility between different tissues of the human body. The technology is highly sensitive to venules, blood components, calcification, and iron deposition and is extremely good at identifying cerebral hemorrhage, especially intracerebral microhemorrhages (with a very high detection rate). Due to the hardened and less elastic vascular wall of PE patients caused by long-term chronic hypertension or the excessive cerebral perfusion caused by a sharp increase in blood pressure, the arteries are easily ruptured, causing hemorrhage. Intracranial hemorrhage is common in patients with RPLS, with an incidence rate of approximately 10–25% (Burnett et al., 2010; Junewar et al., 2014; Karia et al., 2016). Intraparenchymal hemorrhage and subarachnoid hemorrhage (SAH) are the common types of hemorrhage and may occur simultaneously in 18–30% of hemorrhage cases (Sharma et al., 2010). In a retrospective study by Hefzy et al. (2009) of 151 patients, only 3% had SAH, and 8% had lobar hemorrhage. In the retrospective study of Sharma et al. (2010), among 263 RPLS patients, 40 had intracranial hemorrhage, and 5.3% had sulcal SAH. Intracranial hemorrhage may be secondary to anticoagulation therapy and endogenous coagulopathy but is not related to blood pressure. Using SWI-MRI, Mckinney et al. (2012) found that 19.4% of the RPLS patients had intraparenchymal hemorrhage, and 6% had SAH. Even when the lesions in the T2WI sequence completely regressed, tiny hemorrhagic foci were still visible on SWI, so it is necessary to perform follow-up SWI to confirm that the morphological changes are completely resolved.

#### 3.2.4. Diagnostic value of IVIM and MRS for RPLS

Intravoxel incoherent motion is a non-invasive MRI technique for assessing perfusion fraction and edema that can assess blood perfusion at the capillary level without the need for contrast agents. Recent studies have used IVIM to observe that the cerebral blood volume and blood flow indices of PE patients are lower than those of normal pregnant women and non-pregnant women (Yuan et al., 2016; Nelander et al., 2018).

Magnetic resonance spectroscopy can analyze the metabolism of the central nervous system, which is helpful to study the pathogenesis of RPLS at the cellular level (Yuan et al., 2016). MRS can be used to analyze the metabolism of brain lesions in RPLS patients. A prospective observational study of non-pregnant healthy women, healthy pregnant women, and women with PE showed that the *N*-acetylaspartate (NAA)/choline (Cho) ratio gradually increased while the Cho value decreased during healthy pregnancy and that the PE patients had significantly lower NAA/Cho ratios and higher Cho values than healthy pregnant women (Sheikh-Bahaei et al., 2020), which indicated that compared with healthy pregnant women, PE patients had cerebral ischemia. Eichler et al. (2002) found extensive metabolic abnormalities in both brain lesions and adjacent normal brain tissue in patients with RPLS, including increased acetylcholine and creatine levels and mildly decreased NAA levels. A follow-up MRS after 2 months of treatment showed that the metabolic level had returned to normal, confirming the reversibility of RPLS (Eichler et al., 2002).

#### 3.2.5. Related studies of MRI-based oxygen extraction fraction values in RPLS

Magnetic resonance imaging-based OEF mapping uses the phase and amplitude data from QSM-MRI and the amplitude data in QSM and quantitative blood oxygen level-dependent magnitude to calculate the OEF values. This technology can non-invasively and quantitatively measure the functional status of cerebral oxygen metabolism, which is helpful to explore and evaluate the potential impact of changes in cerebral oxygen metabolism on brain function and cognitive behavior. In a recent study, Yang et al. (2022) used MRI-based OEF values to explore functional imaging indicators of cerebral oxygen metabolism in PE patients and to study the interaction between imaging indicators and central nervous system symptoms. They found that the mean OEF values of the thalamus, caudate nucleus, putamen, globus pallidus, and substantia nigra of PE patients were significantly higher than those of healthy pregnant women and non-pregnant healthy women (Yang et al., 2022), which may be due to a compensatory mechanism to overcome severe hypoxia and maintain cerebral oxygen metabolism. The significant changes in cerebral OEF values of pregnant women can be measured by non-invasive MRI-based OEF mapping to determine their cerebral oxygen metabolic status, which can become an important diagnostic basis for timely clinical intervention, thus providing theoretical support for the effective prevention of RPLS.

Functional magnetic resonance imaging (fMRI) is a class of imaging methods developed to demonstrate regional, time-varying changes in brain metabolism. The popularity of fMRI derives from its widespread availability, non-invasive nature, relatively low cost, and good spatial resolution. Increasingly, fMRI is being used as a biomarker for disease, to monitor therapy, or for studying pharmacologic efficacy. With the rapid development of medical imaging technology, some other functional MRI technologies have gradually emerged, including structural magnetic resonance imaging, diffusion tensor imaging, diffusion kurtosis imaging, resting-state functional connectivity MRI, and neurite orientation dispersion and density imaging. However, fMRI has some limitations, such as, resolution in fMRI is limited primarily by SNR because of the necessity for rapid acquisition of time series information, besides, temporal resolution is also affected by hemodynamic response time (Glover, 2011). In a word, although these emerging technologies have not been widely used in routine imaging examinations of RPLS patients, we believed that they will have increasingly more applications in the imaging diagnosis of RPLS as they become more developed.

In summary, although the pathophysiological mechanism of RPLS is still unclear, it is certain that most RPLS cases are reversible after timely and effective diagnosis and treatment. If the disease is allowed to progress, it can cause irreversible neurological damage and severe sequelae. Therefore, timely brain CT or MRI examinations for RPLS patients is particularly important for the early detection and diagnosis of RPLS lesions. Notably, multimodal brain MRI can facilitate the comprehensive evaluation of the degree of brain damage in RPLS patients, provide a theoretical basis for clinical diagnosis and treatment, and help monitor disease progression, improve prognosis, and protect maternal and fetal health.

## Author contributions

NZ and LY wrote the main manuscript text. AH, YW, and GZ collected and organized literatures. TC and YYW revised the manuscript text. All authors reviewed the manuscript and approved the submitted version.
